# Effect of NAC treatment and physical activity on neuroinflammation in subchronic Parkinsonism; is physical activity essential?

**DOI:** 10.1186/s12974-018-1357-4

**Published:** 2018-11-26

**Authors:** Ana-Luisa Gil-Martínez, Lorena Cuenca, Consuelo Sánchez, Cristina Estrada, Emiliano Fernández-Villalba, María Trinidad Herrero

**Affiliations:** 1 Department of Human Anatomy and Psychobiology, Clinical and Experimental Neuroscience Group (NiCE-IMIB), Institute for Aging Research, School of Medicine, University of Murcia, Murcia, Spain; 2 Biomedical Research Institute of Murcia (IMIB-Arrixaca), Campus of Health Sciences, University of Murcia, Murcia, Spain

**Keywords:** Parkinsonism, Neuroinflammation, Oxidative stress, Physical activity, Microglia, Astrocytes, S100b

## Abstract

**Background:**

Neuroprotective strategies are becoming relevant to slow down dopaminergic cell death and inflammatory processes related to the progressive neurodegeneration in Parkinson’s disease (PD). Interestingly, among others, physical activity (PA) or anti-oxidant agents (such as *N*-acetyl-L-cysteine, NAC) are common therapeutic strategies. Therefore, this study aims to analyze if there is a synergistic effect of physical activity along with NAC treatment on dopaminergic degeneration and neuroinflammatory response in a 1-methyl-4-phenyl-1,2,3,6-tetrahydropyridine (MPTP)-induced Parkinsonism model after subchronic intoxication.

**Methods:**

To ascertain this possibility, 48 8-week-old male mice (C57BL/6 strain) were used. Twenty four of them were placed individually in cages where voluntary physical activity was automatically monitored during 30 days and were divided into groups: (i) control; (ii) NAC; (iii) MPTP, and (iv) MPTP+NAC. The other 24 mice were divided into the same four groups but without physical activity.

**Results:**

The data collected during the treatment period showed that there was an overall increase in the total running distance in all groups under physical activity, including Parkinsonian animals. However, the monitoring data per day showed that the activity routine by MPTP and MPTP+NAC groups was disrupted by alterations in the circardian rhythm because of MPTP intoxication. Results from post-mortem studies in the substantia nigra pars compacta (SNpc) showed significant decrease in the number of TH+ cells in all MPTP groups. Moreover, TH+ expression in the striatum was significantly decreased in all MPTP groups. Thus, PA + NAC treatment do not protect dopaminergic neurons against a subchronic intoxication of MPTP. Regarding glial response, the results obtained from microglial analysis do not show significant increase in the number of Iba-1+ cell in MPTP+NAC and MPTP+PA + NAC. In the striatum, a significant decrease is observed only in the MPTP+NAC group compared with that of the MPTP group. The microglial results are reinforced by those obtained from the analysis of astroglial response, in which a decrease in the expression of GFAP+ cells are observed in MPTP+NAC and MPTP+PA + NAC compared with MPTP groups both in the SNpc and in the striatum. Finally, from the study of the astroglial response by the co-localization of GFAP/S100b, we described some expression patterns observed based on the severity of the damage produced by the MPTP intoxication in the different treated groups.

**Conclusions:**

These results suggest that the combination of physical activity with an anti-oxidant agent does not have a synergistic neuroprotective effect in the nigrostriatal pathway. Our results show a potential positive effect, only due to NAC treatment, on the neuroinflammatory response after subchronic MPTP intoxication. Thus, physical activity is not essential, under these conditions. However, we believe that physical activity, used for therapeutic purposes, has a beneficial long-term effect. In this line, these results open the door to design longer studies to demonstrate its promising effect as neuroprotective strategy.

**Electronic supplementary material:**

The online version of this article (10.1186/s12974-018-1357-4) contains supplementary material, which is available to authorized users.

## Main points

Synergistic effect of combined treatment based on physical activity and NAC administration (i) is not enough to decrease dopaminergic neuronal death after MPTP intoxication in SNpc and striatum, and (ii) NAC treatment significantly decrease glial response in subchronic MPTP administration.

## Background

Chronic neuroinflammation and oxidative stress processes are features underlying the development and progression of neurodegenerative disorders. In particular, Parkinson’s disease (PD) is defined by a clinical picture that consists in impaired motor functions and cognitive deficits caused by the progressive loss of dopaminergic neuronal cells in the substantia nigra pars compacta (SNpc) that leads to the depletion of dopamine fibers in the striatum [1, 2] and other catecholaminergic systems [3]. Since the current pharmacological treatments for PD are being controlled in order to reduce motor symptoms [4], new studies are focused on the design of non-pharmacological strategies to avoid side effects caused by drug administration [5].

In this line, physical activity (PA) and exercise attenuate the activation of inflammatory processes in neurological diseases [6]. Although physical activity is defined as “any corporal movement produced by skeletal muscles that requires energy burning” and, exercise as “planned physical activity focused on improving physical abilities” [7], both have similar overall beneficial impact on the body (cardiovascular system, metabolic function, or muscle tone). Additionally, PA can take part in brain functions by improving mood, mental health, and cognitive activity by immune system-mediated effects [8]. Additionally, regular and moderate physical activity maintains the activation of glial cells within a healthy range, which may reduce or delay the progression of brain neurodegeneration [9–14].

Oxidative stress is also a common hallmark in the pathogenesis of PD [15]. It has been described that environmental toxins, such as 1-methyl-4-phenyl-1,2,3,6-tetrahydropyridine (MPTP), contribute to Parkinsonism by increasing oxidative stress in dopaminergic neurons by mitochondrial dysfunction [16]. Several therapeutic strategies, based on antioxidants agents, have been designed to prevent the impact of oxidative processes on the development and progression of neurodegenerative disorders [17]. *N*-acetyl-L-cysteine (NAC) is a glutathione precursor currently tested for its neuroprotective properties in PD patients ( [18] Identifier: NCT01470027). NAC is a well-stablished antioxidant drug that reduces the loss of dopaminergic neurons and protects against MPTP intoxication in mice [19].

However, since the physical activity and NAC do not protect the 100% of the dopaminergic cells after MPTP intoxication, we attempted to devise a combined therapy based on NAC, as an anti-oxidant agent, and physical activity, as a non-pharmacological treatment. Through this strategy, we proposed to analyze the possible synergistic effect on dopaminergic neuronal death and on glial activation after MPTP subchronic intoxication in mice.

## Methods

### Animals

The study was performed on 48 adult male C57BL/6N mice (3 months of age, weight 25–28 g) purchased from Charles River Laboratories (Charles River Laboratories Inc., Barcelona, Spain). Animals were housed in a room with regulated temperature (20 ± 2 °C) on a 12-h light/dark cycle, with ad libitum access to food and water. All experiments were performed according to the European Directive 2010/63/UE and the Spanish RD/53/2013 for the protection of animals used for experimentation and other scientific purposes. All the experimental methods and procedures were approved by Institutional Committee on Animal Ethics of the University of Murcia (REGA ES300305440012).

### Experimental design and treatments

**Fig. 1:**
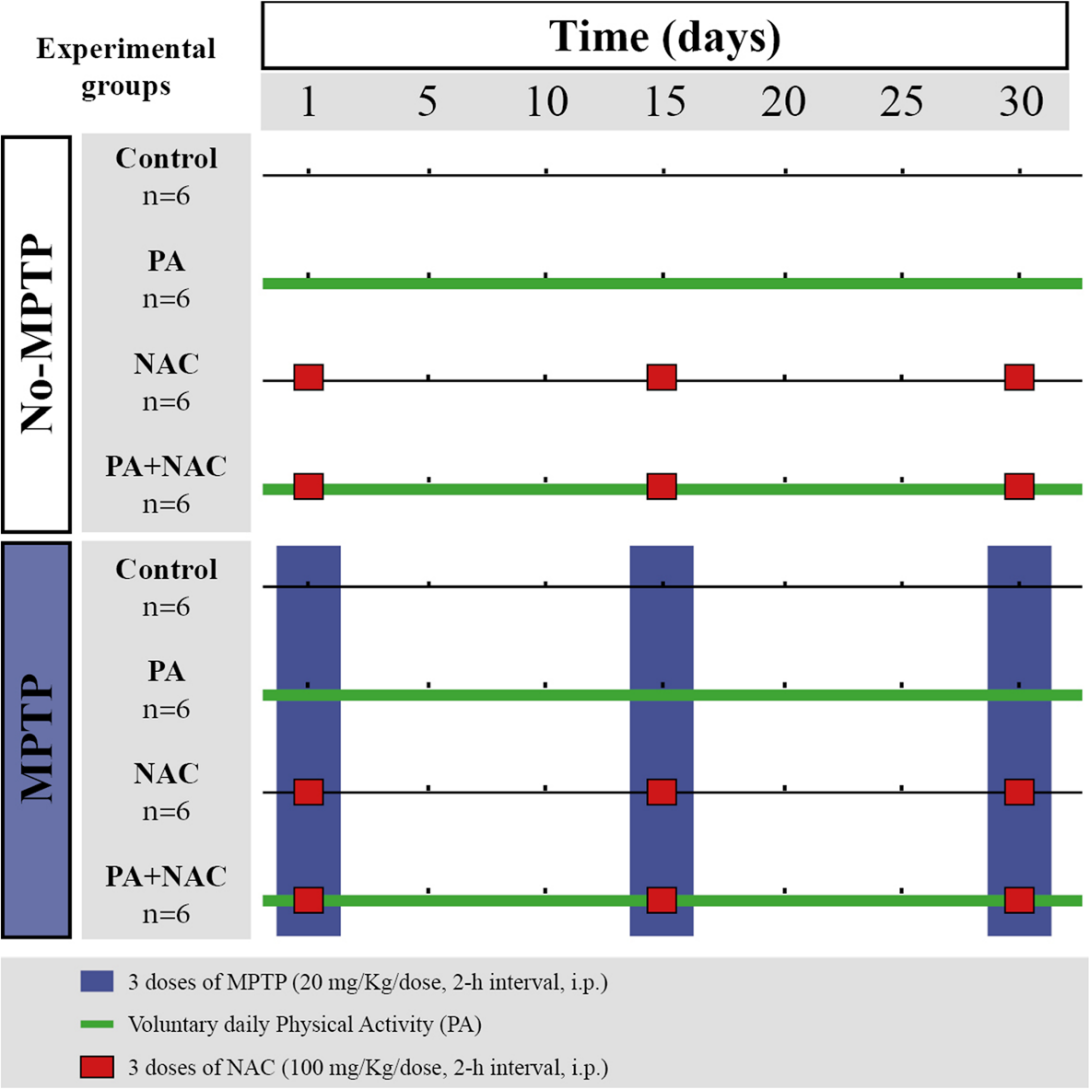
Scheme of the experimental design with the animal distribution and treatments information

All animals were randomly distributed into two main groups (No-MPTP and MPTP), and these turned into 4 other subgroups (*n* = 6, each) that received their respective treatment: (i) control; (ii) physical activity (PA); (iii) *N*-acetyl-L-cysteine (NAC), and (iv) PA + NAC (Fig. 1). All in vivo experiments lasted 30 days in which the treatments were performed, and PA was automatically recorded since day 1 after the first MPTP administration until day 30. PA animals were subjected to daily voluntary physical activity by rotating wheels installed in their individual cages (from days 0 to 30). NAC animals received *N*-acetyl-L-cysteine (NAC, Sigma) (100 mg/kg/dose, 3 doses, 2-h interval) 30 min prior to each MPTP injection at days 0, 15, and 30 [19].

### MPTP administration regime

Parkinsonism was induced to MPTP groups via intraperitoneal (i.p.) injections of 1-Methyl-4-phenyl-1,2,3,6-tetrahydropyridine hydrochloride (MPTP, Sigma-Aldrich) (20 mg/Kg/dose, 3 doses, 2-h interval), dissolved in saline, over a day at days 0, 15 and 30 [20, 21]. After MPTP intoxication, mice remained isolated in a room for 48 h following the safety protocol established for MPTP treatment [22]. No-MPTP animals were treated with vehicle (saline) with the same protocol followed for MPTP injections.

### Physical activity monitoring

**Fig. 2:**
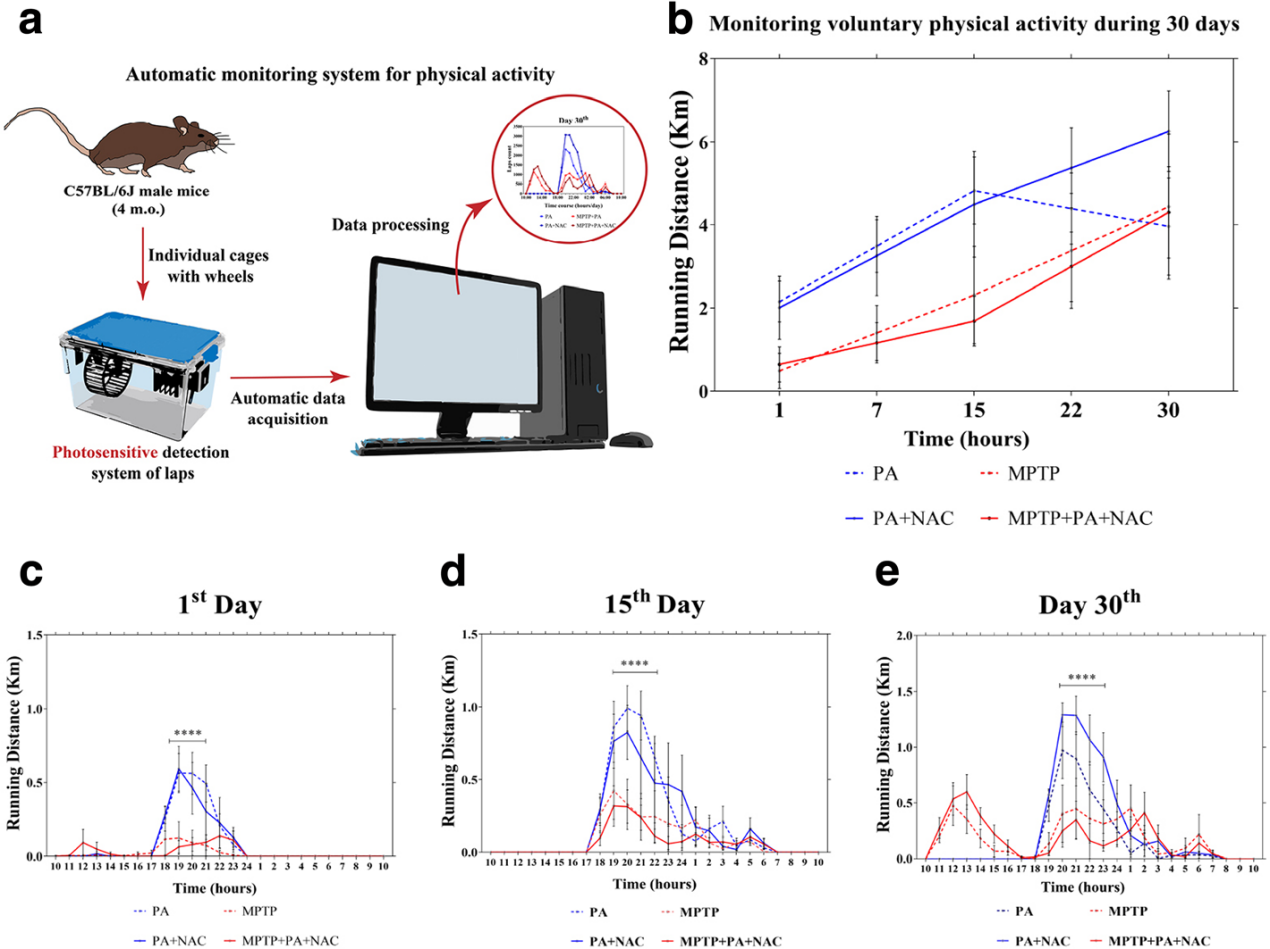
Monitorization of the daily voluntary physical activity. **a**) Scheme of the methodology for the automatic monitoring system for physical activity. **b**) Total running distance (Km) of the different experimental groups during the experiment. It can be observed that No-MPTP mice reach a stable activity on day 15 of the treatment and MPTP treated mice peaked more slowly. **c**–**e** Total running distance (km) through 24 h of the different groups on day 1 (**c**), day 15 (**d**), and 30 (**e**)

Analysis of PA was carried out by a monitored system which measured the complete laps given by each mouse on the wheel per minute during 24 h. A reflective sticker was placed on each wheel, so that the count of laps was based in the reflecting point detected by a photosensitive sensor. This evaluation system aimed to observe the development and progression of the symptoms of both intoxicated and control mice (Fig. 2a). The results from the physical activity monitorization are expressed as running distance in km.

### Tissue collection and specific staining

48 h after the last MPTP injection, mice were intraperitoneally anesthetized with a mixture of Xylazine (50 mg/kg, Xilagesic 2%, Calier Laboratories) and Ketamine (Imagene 50 mg/kg, Merial) mixture at a ratio of 1:1. The brains were immediately removed and fixed with 4% paraformaldehyde in phosphate-buffered saline (PBS, 0.1 M, pH 7.4) and stored at 4 °C. After 48 h, samples were washed with PBS and absolute ethanol and were embedded in paraffin blocks. The striatum and mesencephalon were cut in coronal sections (thickness 7 μm) on a microtome (Thermo Scientific HM 325 Rotary Microtome, Thermo Fisher Scientific).

Series of sections were stained with tyrosine hydroxylase (TH) (mouse polyclonal antibody), ionized calcium-binding adapter molecule 1 (Iba-1) (rabbit polyclonal antibody), glial fibrillary acid protein (GFAP) (mouse polyclonal antibody), and (low molecular) calcium-binding protein b (S100b) (rabbit monoclonal antibody).

### Immunohistochemistry and immunofluorescence

*For DAB detection*. The sections were deparaffinized in xylene, rehydrated in a decreasing ethanol gradient (100%, 95%, and 80%) and distilled water. Antigen retrieval was performed with citrate buffer (10 mM citric acid, pH 6.0) for 30 min at 95 °C with constant shaking. Endogenous peroxidase activity was inhibited with 0.3% H_2_0_2_ for 20 min and non-specific Fc binding sites were blocked with 10% goat serum for 1 h. The sections were incubated overnight (4 °C, constant shaking) with primary antibody diluted (Table 1) in TBS 1× (0.1 M, pH 8.4) containing 1% goat serum and 0.5% Triton X-100. The sections were incubated for 30 min in secondary antibody diluted (Table 1) in TBS 1×. Binding of antibody was detected with avidin-biotin conjugated to peroxidase (ABC Elite Kit, Vectastain, Vector Labs). The sections were dehydrated in graded ethanol series (80%, 95%, and 100%) and in xylene before being cover-slipped [20].

**Table 1:** Dilutions for primary and secondary antibodies used for immunohistochemistry and immunofluorescence

Primary antibodies, code	Host	IHQ/IF	Dilution, incubation time	Secondary antibodies, code	Dilution, incubation time
Anti-TH	Mouse, MAB318, Millipore	IHQ	1:500, o.v.	Anti-IgG mouse (Biotinylated), BA-9200	1:250, 1 h
		IF	1:500, o.v.	Double Labeling Kit, DK8818^a^	30 min
Anti-Iba1	Rabbit, B178846, Abcam	IHQ	1:1000, o.v.	Anti-IgG rabbit (Biotinylated) BA-1000	1:250, 1 h
		IF	1:1000, o.v.	Double Labeling Kit, DK8818^a^	30 min
Anti-GFAP	Mouse, MAB360, Millipore	IHQ	1:500, o.v.	Anti-IgG mouse (Biotinylated) BA-9200	1:250, 1 h
		IF	1:500, o.v.	Double Labeling Kit, DK8818^a^	30 min
Anti-S100b	Rabbit, AB52642, Abcam	IF	1:500, o.v.	Double Labeling Kit, DK8818^a^	30 min

^a^Vectafluor Duet Immunofluorescence Double Labeling Kit, DyLight 488 anti-Rabbit (green)/DyLight 594 Anti-Mouse (red)

*For immunofluorescence detection.* The sections were treated with citrate buffer (10 mM, citric acid, pH 6.0) for 1 h at 95 °C and non-specific Fc binding were blocked with 2.5% goat serum for 1 h. The sections were incubated overnight (4 °C, constant shaking) with primary antibody diluted (Table 1) in TBS 1× containing 2.5% goat serum and 0.5% Triton X-100. The sections were incubated for 30 min in darkness in secondary antibody ready-to-use kit (Double Labeling Kit, Vector Laboratories). The samples were assayed (Vectashield Antifade Mounting Medium, Vector Laboratories).

### Quantification and stereological analysis

The nigrostriatal pathway was defined according to anatomical coordinates of the mouse brain atlas [23] and using the same protocol reported by J. Blesa and colleagues [24] but specifically adjusted to mouse brain [25]. Quantification of DAB-labeled cells were carried out throughout the midbrain [from − 2.06 mm to − 3.80 relative to Bregma], and dopaminergic striatal terminals [from 0.26 mm to 0.02 mm relative to Bregma] was performed on series of eight coronal sections from each animal. All figures for immunohistochemical analysis were obtained by Hall 100 ZEISS optical microscope with an Axiocam ZEISS digital camera.

#### Quantification of TH-positive (TH+) cells

In SNpc, the total number of TH+ cells was determined using the × 20 objective by counting the number of stained neurons (nucleus were used as counting unit) divided by the delineated area of the SNpc expressed as TH+ cells/mm^3^. In the striatum, optical density of TH+ innervations was measured in digitalized images using the NIH Image software (ImageJ; NIH, Bethesda, MD, USA). The optical density of DAB signal in each micrograph was taken with the × 40 objective covering the entire rostro-caudal striatum, expressed as area (%)/mean gray value.

#### Quantification of microglial (Iba-1+) cells

Iba-1-positive cells were counted in SNpc using the same protocol established for TH-labeled samples. In the striatum, the microglia analysis was performed using the × 20 magnification, and Iba-1+ cells were counted and expressed as Iba-1+ cells/mm^3^.

#### Quantification of astrocytes (GFAP+)

In SNpc, astrocytic GFAP expression was analyzed with the same protocol established for TH-labeled samples. In the striatum, the astroglial GFAP expression was carried out using the × 20 objective for counting GFAP+ cells and expressed as GFAP+ cells/mm^3^.

### Confocal analysis and 3D reconstruction

The brain sections were analyzed using Leica TCS-SP8 confocal microscope with the ×40 oil objective (PL APO 40X/1.3 AN) and the × 63 glycol objective (PL APO 63X/1.3 AN) and Leica Application Suite X (LAS X, Leica Microsystems). After the immunostaining, confocal images were obtained in sequential acquisition to avoid cross-reactions or photobleaching of fluorophores during long-lasting manual mode. The images were not post-processed for the analysis. Series of sections were obtained by defining the upper and lower limits using the Z position covering 20 mm of thickness of the brain sample with 0.5 mm thickness per optical section. We show higher magnification images to observe with better detail that the microglial and astroglial morphological features and other aspects, which were not due to invasion or cross-reaction of the fluorescence from other channels. FIJI-ImageJ software was used for the composition of three-dimensional reconstructions from the stacks of confocal images.

### Data and statistical analysis

Data were presented as mean ± SD and were analyzed by means of a two-way analysis of variance test (two-way ANOVA) with a Sidak post hoc analysis for multiple group comparisons. The null hypothesis was rejected at a significance level of 0.05 so that differences with a value of *p* < 0.05 were considered significant and with a value of *p* < 0.01 were considered as statistically very significant. All statistical analyses were conducted using GraphPrism7 software (GraphPad Software Inc.). A detailed description of the statistical analysis can be found in the Additional file 1.

### Figures and artwork

Schematics, tables, and vector graphics were created using Adobe Illustrator CS6, and figures are arranged and compiled using Adobe InDesign CS6.

## Results

### Voluntary PA increased through the experiment in both No-MPTP and MPTP groups

Voluntary physical activity was automatically monitorized during 30 days recording the number of laps performed by each mice expressed as running distance. In Fig. 2b, it is shown that the total running distance increased in all groups since the start of the experiment. A two-way ANOVA (time × groups) was performed and the results indicated a significant main effect for Time [*F*(4, 80) = 25.61, *p* < 0.0001] but not for Groups [*F*[3, 20] = 25.61, *p* < 0.0001]. Following this data, we performed a simple effects within columns. Both PA and PA + NAC groups significantly increase (*p <* 0.05) the running distance from day 15 compared with day 1. In addition, physical activity was also significantly increase for Parkinsonian mice since the beginning but with a shorter mean running distance compared with No-MPTP animals.

In Fig. 2c–e, the monitorization of the physical activity over 24 h in different days is shown. The data was analysis by repeated measures two-way ANOVA (time × groups) that revealed a significant interaction on day 1 [*F*(72, 480) = 3.337, *p* < 0.0001], day 15 [*F*(72, 480) = 2.002, *p* < 0.0001], and day 30 [*F*(72, 480) = 4.412, *p* < 0.0001]. Following this data, we proceeded to analyze simple effects within columns with a Sidak’s as multiple comparisons post hoc test. On day 1, the most significantly peak of activity was registered from 18:00 to 21:00 performed by PA (*p* < 0.0001) and PA + NAC (*p* < 0.0001). For MPTP intoxicated mice there was no significant peak of physical activity registered. On day 15, it is repeated the same pattern of significant peak of physical activity from 18:00 to 22:00 performed by PA (*p* < 0.0001) and PA + NAC (*p* < 0.0001) but not significant peak of running distance was performed by MPTP intoxicated mice. Finally, on day 30, the significant peak of physical activity was performed from 19:00 to 23:00 by PA (*p* < 0.0001) and PA + NAC (*p* < 0.0001) and not significant peak of running distance for Parkinsonian animals. These data show that the total running distance over 1 day does not follow a normal circardian rhythm in MPTP groups compared to the No-MPTP groups, which results in a different activity behavior because of MPTP intoxication.

### PA + NAC treatment is not enough to protect DA neurons under subchronic MPTP intoxication

**Fig. 3:**
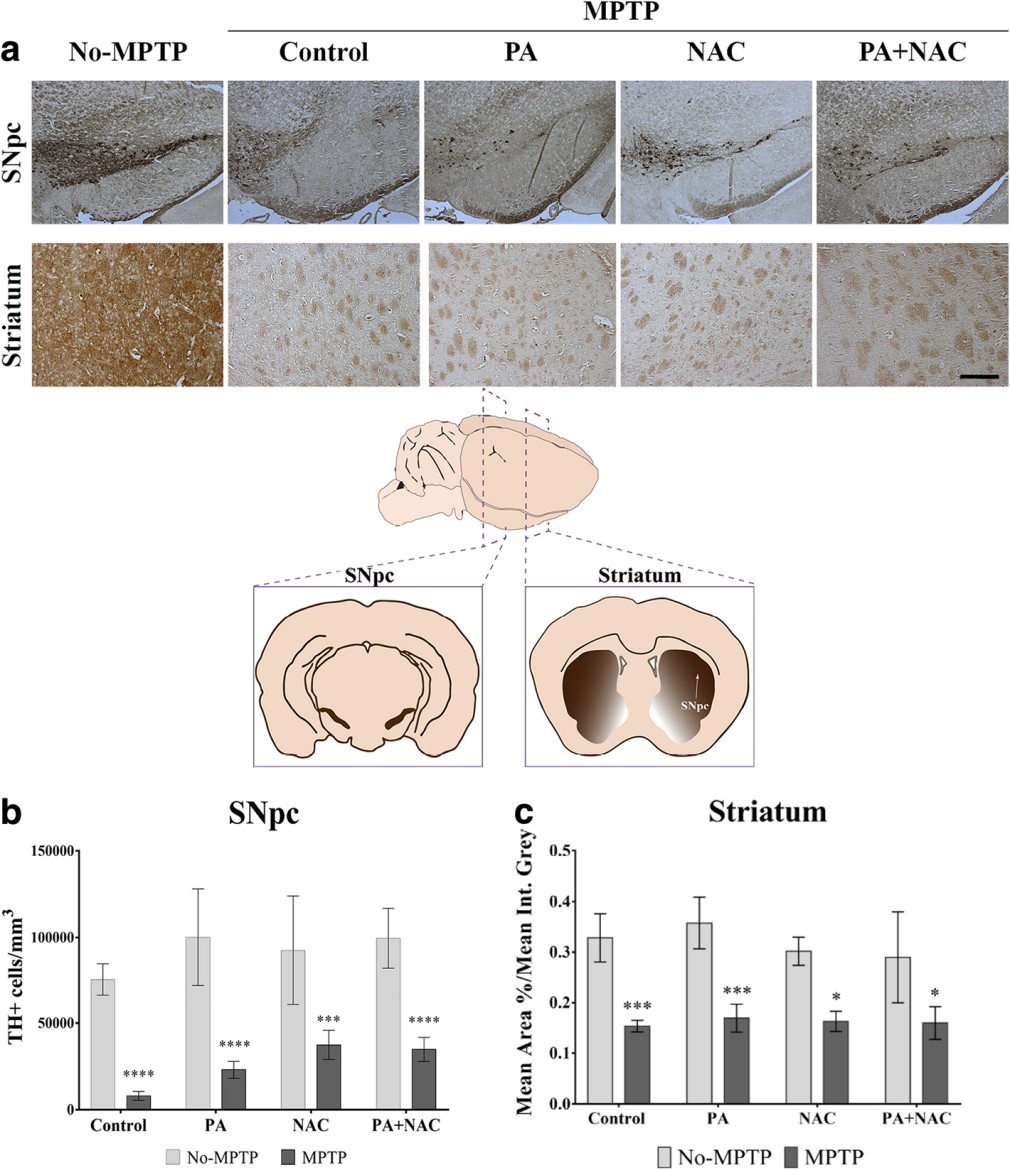
Quantification of TH+ expression in SNpc and striatum. **a** Representative images from TH immunolabeling in SNpc and striatum (magnification × 10, scale bar = 100 μm). **b** Quantification of TH+ cells/mm^3^ in SNpc. Results show that MPTP (****p < 0.0001), MPTP+PA (****p = 0.0001), MPTP+NAC (***p = 0.0006), and MPTP+PA + NAC (****p < 0.0001) have a significant decrease of TH+ cells/mm^3^ compared to their No-MPTP groups. **c** Measurement of TH+ expression in striatal fibers. All MPTP mice have a significant decrease compared with their respective control groups: MPTP (***p = 0.0007), MPTP+PA (***p = 0.0004), MPTP+NAC (*p = 0.0143), and MPTP+NAC + PA (*p = 0.0396)

It has been described that in PD, there is a dopaminergic neuronal loss in the SNpc and in the neuronal striatal innervations. In order to study the possible neuroprotective effect of PA, NAC, and, mainly, the combined PA + NAC treatment on dopaminergic neuronal death caused by subchronic MPTP-injections, we assessed immunohistochemistry for TH expression in SNpc and striatum (Fig. 3). All Parkinsonized mice suffered a significant loss of TH expression in dopaminergic neurons compared to their respective No-MPTP groups. A two-way ANOVA (treatments × Parkinsonism) was performed, and the results indicated a significant main effect for treatments [*F*[3, 31] = 5.1420, *p* =0.0053] and for Parkinsonism [*F*[1, 31] = 156.70, *p* <0.0001]. There was no significant interaction between these two variables [*F*[3, 31] = 0.7234, *p* = 0.5457] so that they cause an overall main effects (Fig. 3b).

In the same way, dopaminergic striatal innervation was analyzed by optical density measurements of TH-stained slices. The MPTP intoxication induced a significant main effect [*F*[1, 31] = 82.70, *p* < 0.0001] in all the groups. However, there is no effect overall by treatments variable [*F*[3, 31] = 0.9184, *p* = 0.4469], and the interaction is considered not significant [*F*[3, 31] = 0.6373, *p* = 0.5983] (Fig. 3c). These data demonstrate that there is a very significant main effect of MPTP intoxication in all groups under subchronic administration that it is not enough by the administration of NAC or/and physical activity.

### Effect of PA + NAC treatment on Iba-1 expression in the SNpc and striatum after MPTP intoxication

**Fig. 4:**
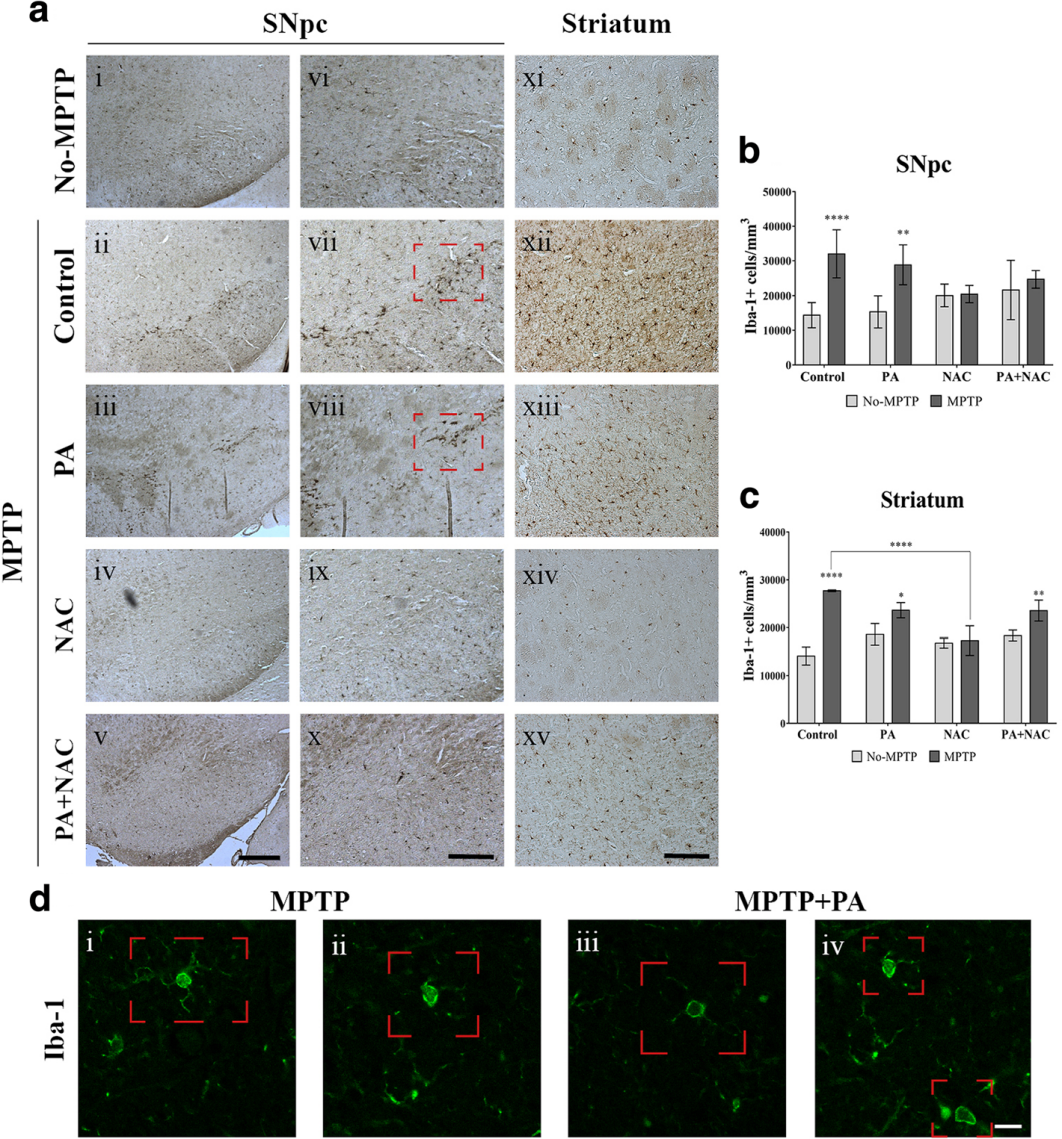
Microglial cells quantification in the nigrostriatal pathway. **a** Representative photomicrographs of Iba-1+ expression in SNpc and striatum (magnification × 10 (i–v) and × 20 (vi–xv), scale bar = 100 μm and 50 μm, respectively). **b** Iba-1+ cells density in the SNpc is significantly increase in MPTP (****p < 0.0001) and MPTP+PA (**p = 0.0040) groups compared to their No-MPTP groups. **c** In the striatum, microglial cells expression were significantly increase in MPTP (****p < 0.0001), MPTP+PA (*p = 0.0348), and MPTP+PA + NAC (**p = 0.0082). Interestingly, it can be observed that significant decrease is found in MPTP+NAC (****p < 0.0001) compared to MPTP group. **d** Representative micrographs from immunofluorescence under high magnification showing Iba-1+ cells in the SNpc of MPTP (i–ii) and MPTP+PA (iii–iv) groups (magnification × 63, scale bar = 25 μm)

We studied microglial activation by analysis for Iba-1 labeling, a specific microglial marker which does not cross-react neither with neurons nor astrocytes (Fig. 4). In the SNpc, no significant increase of microglial cells is observed in untreated mice and in MPTP+NAC and MPTP+PA + NAC groups while MPTP (*p* < 0.0001) and MPTP+PA (*p* = 0.0040) groups presented a significant increase in the number of Iba-1+ cells compared to their No-MPTP groups (Fig. 4b). According to these results, MPTP (*p* < 0.0001) and MPTP+PA (*p* = 0.0348) mice showed a significant increase in microglial activation in the striatum compared to their No-MPTP groups; this increment can also be observed in the MPTP+PA + NAC group (*p* = 0.0082), although this microglial cells may have adopted a protective role [26] (Fig. 4c). Reinforcing the results obtained by immunohistochemical analysis, it can be observed in Fig. 4d, morphological characteristics, already described for active microglia [27].

### Effect of PA + NAC treatment on GFAP expression in the SNpc and striatum after MPTP intoxication

**Fig. 5:**
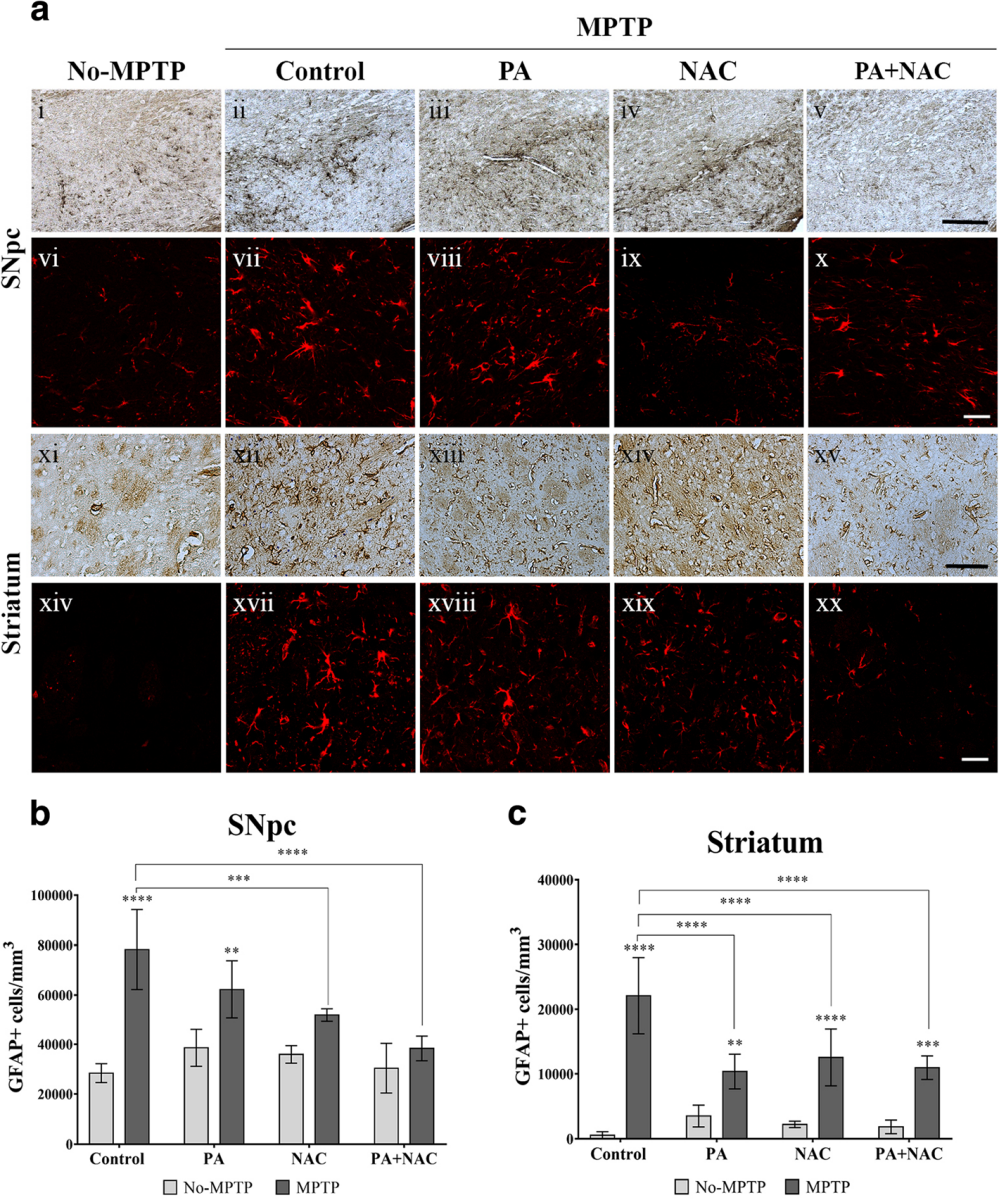
Effect of combined treatment on GFAP+ expression in the SNpc and striatum. **a** Images from immunolabeling of GFAP in the SNpc and in the striatum (magnification × 20 (i–v and xi–xv), scale bar = 50 μm; magnification × 63 (vi–x and xvi–xx, scale bar = 25 μm). **b** GFAP+ cells density in the SNpc is significantly increase in MPTP (****p < 0.0001) and MPTP+PA (***p = 0.0021) groups compared to No-MPTP groups. Interestingly, a significant decrease in GFAP+ expression were found comparing MPTP+NAC (***p = 0.0008 and MPTP+PA + NAC (****p < 0.0001) with MPTP group. **c** In the striatum, MPTP (****p < 0.0001), MPTP+PA (**p = 0.0060), MPTP+NAC (****p < 0.0001), and MPTP+PA + NAC (***p = 0.0002) express significant increase in GFAP+ cells density comparing with their respective No-MPTP groups. As in the SNpc, significant decrease were found between MPTP+PA (****p < 0.0001), MPTP+NAC (***p = 0.0002), and MPTP+PA + NAC (****p < 0.0001)

Activated astrocytes are characterized by higher GFAP expression levels and increased cell body size (hypertrophy) in the affected brain regions [28]. Quantification of GFAP+ cells in the SNpc showed a significant increase in the MPTP (*p* < 0.0001) and in MPTP+PA (*p* = 0.0021) groups compared with their control groups. In relation to the results described above, no significant differences in astrocytes activation were found in MPTP+NAC and MPTP+PA + NAC when compared to the NAC and PA + NAC groups, respectively (Fig. 5b). These findings point out to a decrease in the damage produced by the administration of MPTP when animals are treated with NAC and with the combination of PA + NAC, since a significant decrease in the number of GFAP+ cells is observed in MPTP+NAC (*p* = 0.0008) and MPTP+PA + NAC (*p* < 0.0001) compared to the MPTP-treated group. In the striatum, the number of GFAP+ cells was significantly increased in all MPTP groups compared to their respective No-MPTP groups: MPTP (*p* < 0.0001), MPTP+PA (*p* = 0.0060), MPTP+NAC (*p* < 0.0001), and MPTP+PA + NAC (*p* = 0.0002). However, although there is a significant increase in the number of astrocytes that express GFAP in all MPTP groups when they are compared with their respective control groups, it is interesting to note that this significant increase in MPTP mice is maintained when compared to Parkinsonian animals treated with PA (*p* < 0.0001), NAC (*p* = 0.0002), and PA + NAC (*p* < 0.0001) (Fig. 5c).

### GFAP/S100b co-localization in the SNpc and striatum: different astrocyte profiles based on groups treatment

In our study, we described profiles of expression (S100b/GFAP) that were repeated among the individuals of the same treatment group.

**Fig. 6:**
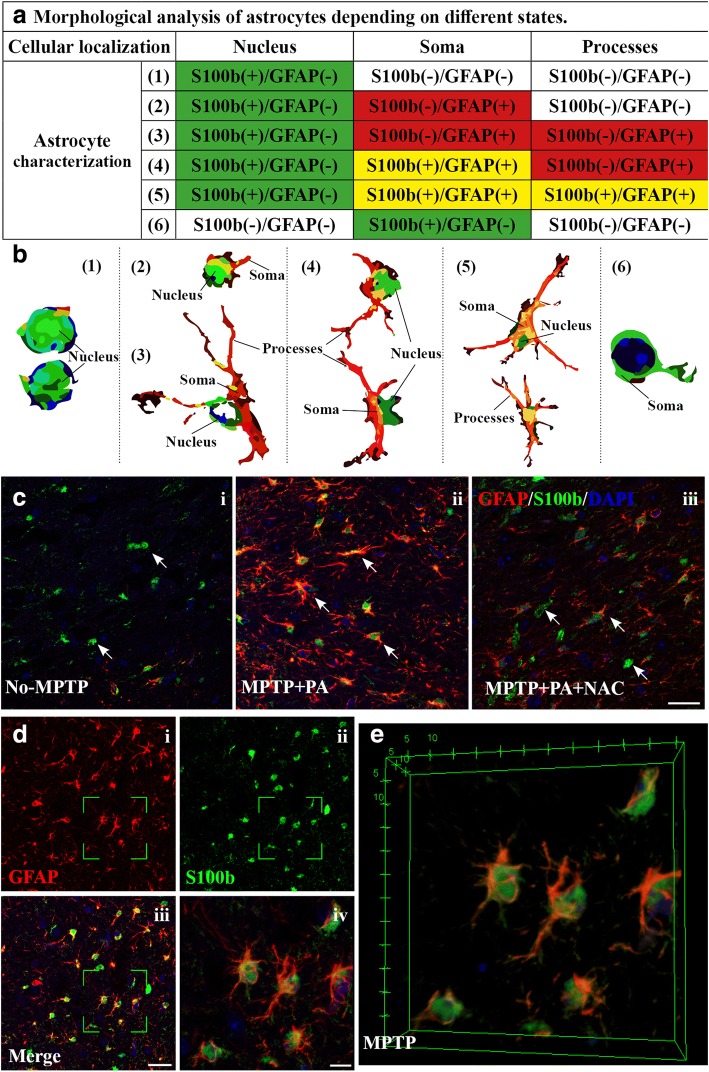
Description of the observed cellular location profiles of GFAP and S100b in astrocytes. **a** Descriptive table with the details of the different cellular location profiles. **b** Cartoons from 1 to 6 illustrate the different location profiles described in the table. **c** Representative images of the experimental groups: No-MPTP, MPTP+PA, and MPTP+PA + NAC where these expression profiles are observed. **d** Frame showing the separate channels, GFAP (i) and S100b (ii), merge (iii) and 3D projection from z-stack images (iv) in which showed a selective area at high magnification. **e** 3D reconstruction images to show how it is observed GFAP, S100b, and colocalization of both proteins. (magnification × 63, zoom set at 1, scale bar = 25 μm)

Thus, we established six profiles of expression: [1] the expression of S100b is only located in the nucleus and GFAP is not expressed; [2] S100b is expressed in the nucleus and GFAP expression is located in the soma; [3] S100b expression is only located in the nucleus whereas GFAP expression is in the soma and in the processes; [4] S100b is located in the nucleus but, in this case, it is found a double-labeled S100b/GFAP in the soma; [5] same characteristics as state 4, but with double-labeled both in the soma and in the processes; and [6] only S100b positive expression in the soma. In all MPTP-untreated mice, mainly profile [1] was found (Fig. 6). In MPTP and MPTP+PA groups, states 4 and 5 were found; the colocalization between S100b and GFAP is what characterizes these two profiles, which, in turn, could be related to a more severe injury after MPTP administration. Finally, MPTP+NAC and MPTP+PA + NAC groups show 2 and 3 expression profiles, which could be translated to an acute damage.

## Discussion

In this study, the regimen of MPTP administration was defined based on the dynamic changes in the nigrostriatal pathways [29]. We stablished three MPTP administrations (3 doses, at 2-h intervals in 1 day) distributed in three time-points. The first administration was performed on day 1 of the treatment and combined with the administration of NAC before each dose of MPTP [19] in the MPTP+NAC group. The established treatment regime was repeated on the second and third weeks (day 15 and day 30, respectively) after the first MPTP administration. Animals were sacrificed 48 h after the last injections because it has been described to be the highest peak of glial activation after MPTP injections [21].

Voluntary physical activity was automatically controlled daily in order to analyze the effect on the MPTP intoxication. The results obtained from the total balance of laps per day in the MPTP-treated mice subjected to daily PA showed a stabilization for activity from the beginning to the end of the treatment. It is important to highlight that time has a significant effect on promoting the increase of PA, implying that over the 3-week experiment, both No-MPTP and MPTP groups were more willing to run. Considerably, data obtained from 24-h monitoring presented significant changes between groups, which were accentuated 48 h after MPTP injections. Thus, the rhythm of activity is different between MPTP and No-MPTP animals, peaks of diurnal and nocturnal activity were observed in the intoxicated groups. These results agree with other studies conducted in Parkinsonized macaques whose motor activities varied throughout the day as a consequence of an alteration of the circadian rhythm caused by the MPTP intoxication [30, 31].

Both PA and NAC have individually showed to significantly decrease the loss of dopaminergic neurons in MPTP-induced Parkinsonian mice [6, 19]. However, our data showed a significant decrease of TH+ expression both in the SNpc and in the striatum in all MPTP groups. The fact that dopaminergic neurons are not protected against MPTP intoxication by the treatment based on NAC and physical activity can be explained by the experimental design. Regimes known as sub-chronic are, indeed, serial MPTP acute insults over days or weeks. The samples were obtained 48 h after the last MPTP administration, so we were analyzing the accumulative damage caused by the serial MPTP injections over 3 weeks. Specifically, in the SNpc there is a significant main effect from the treatments, so it may be potentially important to design long-term studies to analyze the recovery capacity of dopaminergic neurons in Parkinsonian mice treated with PA + NAC.

Several investigations suggest that a sustained inflammatory response is a crucial fact for the progression of neuronal depletion in PD [32]. Inflammation in the central nervous system (CNS) involves the intervention of a specialized response by activated glial cells and oxidative stress mechanisms [33, 34]. These mainly include microglia, the major resident immune cells in the brain, and astrocytes, the supportive glial cells components in neuronal tissue. After an acute injury, glial cells become activated (reactive gliosis) and work to repair the damage and restore cerebral homeostasis. Recent works show that PA has emerged as a potent non-pharmacological component with immune-modifying properties, resulting in an overall whole-body anti-inflammatory effect [6]. Thus, it has been reported that endurance exercise has a neuroprotective effect against dopamine loss and proinflammatory cytokines production by modulating TLR2/MyD88/NF-kB activation, and it also may be implicated in downregulation of a-synuclein [35].

In this work, we analyzed the dynamics interplay between microglia and astroglial cells under different therapeutic conditions in Parkinsonian mice. In the first instance, microglial cells proceed with an anti-inflammatory phenotype with neuroprotective effects [29]. If the initial acute damage is not resolved, a maintained inflammatory response is established and affects surrounding glial cells and neurons promoting a detrimental progression of the disease [36]. In fact, microglial cells’ quantification showed a significant increase in the expression of Iba-1 in all MPTP intoxicated groups, with the exception of Parkinsonian mice treated with NAC and PA + NAC in the SNpc. In the striatum, Iba-1+ expression was significantly increased only in MPTP and MPTP+PA groups compared with their No-MPTP groups, and no significant changes were observed in MPTP+NAC and MPTP+PA + NAC. In the striatum, Iba-1+ cells were significantly increased in MPTP and MPTP+PA compared with their No-MPTP groups.

Reactive astrogliosis is triggered by active microglia [37] or dopaminergic neuronal death [38]. In the SNpc, our data showed that GFAP+ expression was significantly increased in MPTP and MPTP+PA groups while no significant results were found in MPTP+NAC and MPTP+PA + NAC compared to their No-MPTP groups. These data reinforce the results obtained for microglia activation.

Although the literature does not reach a consensus in the description and identification of the morphological characteristics of activated glial cells, some features are undoubted. Activated microglial cells acquire a heterogeneous forms from ramified to amoeboid macrophage-like appearance [39]. In this line, changes in reactive astrocytes depend on the severity of the aggression including progressive alterations in molecular expression, cell hypertrophy, and in severe cases, proliferation and scar formation [40]. In this study, we observed these profiles of morphological changes both in the SNpc and in the striatum in those groups that were more damaged by the MPTP intoxication (Figures 5 (vi–x and xi–xv)).

Finally, we studied S100b, a protein mainly expressed in astrocytes with an important role in the initial phase of brain insults as well as maintenance of glial-mediated pro-inflammatory state. Its release by astroglia varies depending on different factors as TNF-α in degenerative disorders [41]. In the present study, different GFAP and S100b expression profiles were defined based on the observations of the dual immunolabeling. We propose two possible situations regarding immunofluorescence analysis; one of them is the possibility of observing subpopulations of astrocytes that are evidenced by different colocalization profiles of GFAP and S100b [42]. However, our interpretation of the results fits better with a second proposed situation that supports that different colocalization profiles change depending on the severity of the injury. Thus, as it has already been shown, S100b would be released by GFAP+ and GFAP− astrocytes may play an important role in the development of MPTP-induced dopaminergic neurodegeneration in mice [43]. In this study, we mainly found that S100b is located in the nucleus of GFAP− astrocytes in No-MPTP, MPTP+NAC, and MPTP+PA + NAC groups, which showed to be the less affected. These results are in accordance with a previous study [43]. Then, we observed an expression of S100b moving from the nucleus to the soma in both GFAP+ and GFAP− astrocytes in groups from least (MPTP+NAC and MPTP+PA + NAC) to most (MPTP and MPTP+PA) damaged animals by MPTP intoxication, respectively. This data could be especially interesting in the identification of the astrocyte’s activation state and therefore in identifying the degree of severity under a pathological condition.

## Conclusions

These results suggest that the combination of physical activity with an anti-oxidant agent does not have a synergistic neuroprotective effect in the nigrostriatal pathway after subchronic MPTP intoxication. However, our results show a potential positive effect only due to NAC treatment on the neuroinflamatory response both in the SNpc and in the striatum after subchronic MPTP intoxication. Thus, concluding that physical activity, under these conditions, is not necessary. However, we believe that physical activity, used for therapeutic purposes, has a beneficial long-term effect. In this line, these results open the door to design longer studies to demonstrate the promising effect of physical activity as neuroprotective strategy.

## Additional file

Additional file 1: Detailed description of the statistical analysis. (DOCX 234 kb)

## Abbreviations

CNS: Central nervous system
DA: Dopaminergic
GFAP: Glial fibrillary acidic protein
Iba-1: Ionized calcium-binding adapter molecule 1
IR: Immunoreactive
MPTP: 1-methyl-4-phenyl-1,2,3,6-tetrahydropyridine
NAC: *N*-acetyl-L-cysteine
PA: Physical activity
PD: Parkinson’s disease
SNpc: Substantia nigra pars compacta
