# Supplementary material for: Effect of NAC treatment and physical activity on neuroinflammation in subchronic Parkinsonism; is physical activity essential?
Source: J Neuroinflammation. 2018 Nov 26;15:328. doi: 10.1186/s12974-018-1357-4 (PMC6260767; doi:10.1186/s12974-018-1357-4)
Supplement: Supplementary file 1 — Detailed description of the statistical analysis. (DOCX 234 kb) [file 12974_2018_1357_MOESM1_ESM.docx]

**MULTIFACTOR ANOVA INFORMATION**

**Monitorization of the physical activity throughout the 30 days of treatment**

| Table S1. Tabular results from repeated measures two-way ANOVA analysis for the running distance (Km) over 30 days. Data represented in the graph from Figure 2b. | | | | | |
| --- | --- | --- | --- | --- | --- |
|  | **SS** | **DF** | **MS** | ***F* value** | ***p* value** |
| Interaction | 28.40 | 12 | 2.367 | 1.375 | 0.1954 |
| Time | 176.4 | 4 | 44.10 | 25.61 | <0.0001 |
| Groups | 95.64 | 3 | 31.88 | 1.585 | 0.2242 |
| Residual | 137.7 | 80 | 1.722 |  |  |
| SS: sum of squares/ DF: degrees of freedom/ MS: mean square | | | | | |

A two-way ANOVA (Time x Groups) was performed and the results indicated a significant main effect for Time [*F* (4, 80) = 25.61, *p* < 0.0001] and not for Groups [*F* (3, 20) = 1.585, *p* = 0.2242]. The interaction is considered not significant [*F* (12, 80) = 1.375, *p* = 0.1954].

**Monitorization of the physical activity over 24h**

A repeated measures two-way ANOVA was carried out for compare over 24h the monitorization of the physical activity between the different groups. The information concerning ANOVA analysis is collected in table S2.

| Table S2. Tabular results from repeated measures two-way ANOVA analysis for the running distance (Km) over 24h on days after MPTP administrations. Data in graphs in Figure 2c-d. | | | | | |
| --- | --- | --- | --- | --- | --- |
| 1^st^ day | **SS** | **DF** | **MS** | ***F* value** | ***p* value** |
| Interaction | 3.249 | 72 | 0.04513 | 3.337 | <0.0001 |
| Time | 5.890 | 24 | 0.2454 | 18.15 | <0.0001 |
| Groups | 0.5574 | 3 | 0.1858 | 2.65 | 0.0767 |
| Residual | 6.490 | 480 | 0.0701 |  |  |
| 15^th^ day | **SS** | **DF** | **MS** | ***F* value** | ***p* value** |
| Interaction | 6.208 | 72 | 0.0862 | 2.002 | <0.0001 |
| Time | 21.92 | 24 | 0.9134 | 21.20 | <0.0001 |
| Groups | 1.758 | 3 | 0.5858 | 2.436 | 0.0946 |
| Residual | 20.68 | 480 | 0.0431 |  |  |
| 30^th^ day | **SS** | **DF** | **MS** | ***F*** **value** | ***p* value** |
| Interaction | 18.62 | 72 | 0.259 | 4.412 | <0.0001 |
| Time | 28.08 | 24 | 1.170 | 19.96 | <0.0001 |
| Groups | 0.752 | 3 | 0.254 | 0.6446 | 0.5954 |
| Residual | 28.14 | 480 | 0.059 |  |  |
| SS: sum of squares/ DF: degrees of freedom/ MS: mean square | | | | | |

Repeated measures two-way ANOVA (Time x Groups) revealed a significant interaction on day 1^st^ [*F* (72, 480) = 3.337, *p* < 0.0001], day 15^th^ [*F* (72, 480) = 2.002, *p* < 0.0001] and day 30^th^ [*F* (72, 480) = 4.412, *p* < 0.0001]. Following this data, we proceeded to analyze with a Sidak’s as multiple comparisons post-hoc test. On day 1^st^, the most significantly peak of activity was registered from 18:00 to 21:00 performed by PA (*p* < 0.0001) and PA+NAC (*p* < 0.0001). For MPTP intoxicated mice there was no significant peak of physical activity registered. On day 15^th^, it is repeated the same pattern of significant peak of physical activity from 18:00 to 22:00 performed by PA (*p* < 0.0001) and PA+NAC (*p* < 0.0001) but not significant peak of running distance was performed by MPTP intoxicated mice. Finally, on day 30^th^, the significant peak of physical activity was performed from 19:00 to 23:00 by PA (*p* < 0.0001) and PA+NAC (*p* < 0.0001) and not significant peak of running distance for parkinsonian animals.

**Dopaminergic neuronal death**

| **Table S3**. Multifactor ANOVA table for the variables and factors for **TH+ cells/mm^3^ in the SNpc**. Data represented with post-hoc analyses in **Figure 3b.** | | | | |
| --- | --- | --- | --- | --- |
|  | | | | 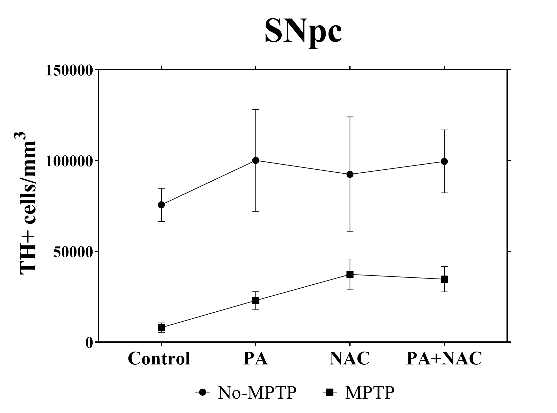 |
|  | DF | F | P |  |
| Interaction | 3 | 0.7234 | 0.5457 |  |
| Treatments | 3 | 5.1420 | 0.0053 |  |
| Parkinsonism | 1 | 156.70 | <0.0001 |  |
| Residual | 31 |  |  |  |
| DF: degrees of freedom; F: *F* value; P: *p* value | | | |  |

A two-way ANOVA (Treatments x Parkinsonism) was performed and the results indicated a significant main effect for Treatments [*F* (3, 31) = 5.1420, *p* = 0.0053] and for Parkinsonism [*F* (1, 31) = 156.70, *p* < 0.0001]. There was no significant interaction between these two variables [*F* (3, 31) = 0.7234, *p* = 0.5457].

| **Table S4**. Multifactor ANOVA table for the variables and factors for **Mean Area %/ Mean Int. Grey in the striatum**. Data represented with post-hoc analyses in **Figure 3c.** | | | | |
| --- | --- | --- | --- | --- |
|  | | | | 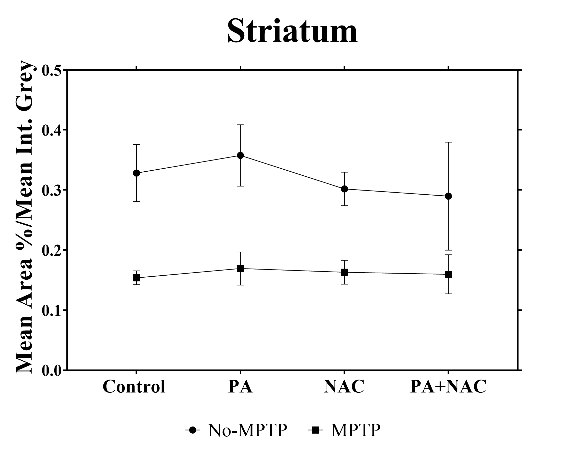 |
|  | DF | F | P |  |
| Interaction | 3 | 0.6373 | 0.5983 |  |
| Treatments | 3 | 0.9184 | 0.4469 |  |
| Parkinsonism | 1 | 82.76 | <0.0001 |  |
| Residual | 31 |  |  |  |
| DF: degrees of freedom; F: *F* value; P: *p* value | | | |  |

A two-way ANOVA (Treatments x Parkinsonism) was performed and the results indicated a significant main effect for Parkinsonism [*F* (1, 31) = 82.76, *p* < 0.0001] and not for Treatments [*F* (3, 31) = 0.9184, *p* = 0.4469]. The interaction is considered not significant [*F* (3, 31) = 0.6373, *p* = 0.5983].

**Microglial activation**

| **Table S5**. Multifactor ANOVA table for the variables and factors for **Iba-1+ cells/mm^3^ in the SNpc**. Data represented with post-hoc analyses in **Figure 4b.** | | | | |
| --- | --- | --- | --- | --- |
|  | | | | 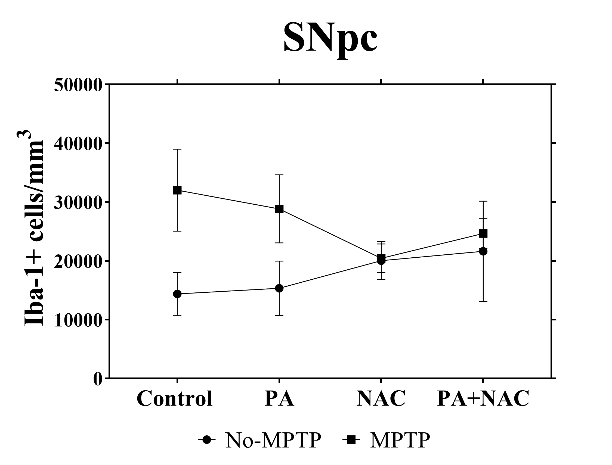 |
|  | DF | F | P |  |
| Interaction | 3 | 6.023 | 0.0024 |  |
| Treatments | 3 | 0.6243 | 0.6049 |  |
| Parkinsonism | 1 | 26.33 | <0.0001 |  |
| Residual | 30 |  |  |  |
| DF: degrees of freedom; F: *F* value; P: *p* value | | | |  |

A two-way ANOVA (Treatments x Parkinsonism) was performed and the results indicated a significant main effect for Parkinsonism [*F* (1, 30) = 26.33, *p* < 0.0001] but not significant for Treatment [*F* (3, 30) = 0.6243, *p* = 0.6049]. The interaction was considered very significant [*F* (3, 30) = 6.023, *p* = 0.0024].

| **Table S6.** Multifactor ANOVA table for the variables and factors for **Iba-1+ cells/mm^3^ in the striatum**. Data represented with post-hoc analyses in **Figure 4d.** | | | | |
| --- | --- | --- | --- | --- |
|  | | | | 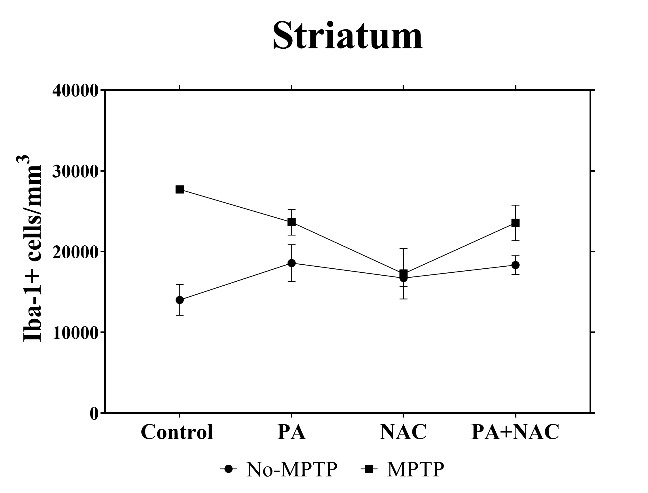 |
|  | DF | F | P |  |
| Interaction | 3 | 12.79 | <0.0001 |  |
| Treatments | 3 | 9.511 | 0.0002 |  |
| Parkinsonism | 1 | 74.87 | <0.0001 |  |
| Residual | 30 |  |  |  |
| DF: degrees of freedom; F: *F* value; P: *p* value | | | |  |

A two-way ANOVA (Treatments x Parkinsonism) was performed and the results indicated a significant main effect for Parkinsonism [*F* (1, 30) = 74.87, *p* < 0.0001] and for Treatment [*F* (3, 30) = 9.511, *p* < 0.0001]. The interaction was considered very significant [*F* (3, 30) = 12.79, *p* = 0.0024].

**Astroglial activation**

| **Table S7**. Multifactor ANOVA table for the variables and factors for **GFAP+ cells/mm^3^ in the SNpc**. Data represented with *post-hoc* analyses in **Figure 5b.** | | | | |
| --- | --- | --- | --- | --- |
|  | | | | 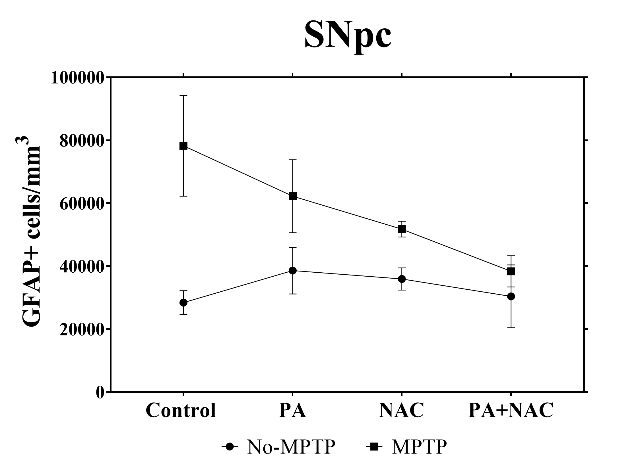 |
|  | DF | F | P |  |
| Interaction | 3 | 11.51 | <0.0001 |  |
| Treatments | 3 | 9.496 | 0.0001 |  |
| Parkinsonism | 1 | 81.09 | <0.0001 |  |
| Residual | 34 |  |  |  |
| DF: degrees of freedom; F: *F* value; P: *p* value | | | |  |

A two-way ANOVA (Treatments x Parkinsonism) was performed and the results indicated a significant main effect for Parkinsonism [*F* (1, 34) = 81.09, *p* < 0.0001] and for Treatment [*F* (3, 34) = 9.496, *p* = 0.0001]. The interaction was considered very significant [*F* (3, 34) = 11.51, *p* < 0.0001].

| **Table S8**. Multifactor ANOVA table for the variables and factors for **GFAP+ cells/mm^3^ in the striatum**. Data represented with post-hoc analyses in **Figure 5c.** | | | | |
| --- | --- | --- | --- | --- |
|  | | | | 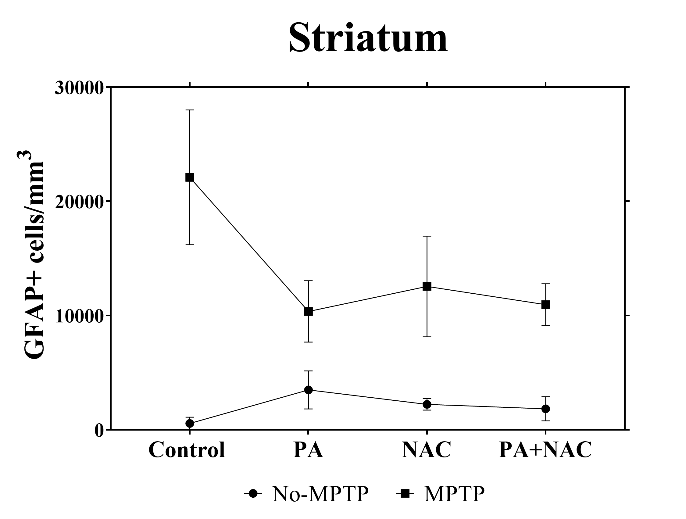 |
|  | DF | F | P |  |
| Interaction | 3 | 13.48 | <0.0001 |  |
| Treatments | 3 | 6.264 | 0.0016 |  |
| Parkinsonism | 1 | 184.1 | <0.0001 |  |
| Residual | 35 |  |  |  |
| DF: degrees of freedom; F: *F* value; P: *p* value | | | |  |

A two-way ANOVA (Treatments x Parkinsonism) was performed and the results indicated a significant main effect for Parkinsonism [*F* (1, 35) = 184.1, *p* < 0.0001] and for Treatment [*F* (3, 35) = 6.264, *p* = 0.0016]. The interaction was considered very significant [*F* (3, 35) = 13.48, *p* < 0.0001].
